# Cesarean Section Scar Ectopic Pregnancy in the Second Trimester: An Underrecognized Complication of Cesarean Deliveries

**DOI:** 10.1155/2021/8888019

**Published:** 2021-02-09

**Authors:** Sharonne Holtzman, Mary Louise Kiernan, Jaimie Huntly, Valentin Kolev, Konstatin Zakashansky

**Affiliations:** Department of Obstetrics, Gynecology and Reproductive Sciences, Icahn School of Medicine at Mount Sinai Hospital, USA

## Abstract

The aim of this paper is to present a case of a cesarean section ectopic pregnancy (CSP) diagnosed in the second trimester and perform a literature review of current guidelines for the management of CSP in the second trimester. This was exempt from the Mount Sinai IRB. This is a case is of a 35-year-old P1122 at 13w4d who presented to our hospital with vaginal spotting and abdominal pain. The patient was found to have a cesarean section ectopic pregnancy with placenta increta. There are no management guidelines for second trimester CSP, and the published material is minimal. A literature review was completed and demonstrated two cases and one case series published on management of existing literature on management of second trimester CSP. Our patient underwent an uncomplicated total laparoscopic hysterectomy with bilateral salpingectomy, bilateral ureterolysis, and cystoscopy. She had an uncomplicated postoperative course and was discharged on postoperative day three with an unremarkable recovery at her two-week postoperative visit.

## 1. Introduction

The high rate of cesarean delivery (CD) in the United States is well documented. The CDC released the CD rate for the year of 2018 as 31.9%, increasing by 54% from the lowest CD rate in 1996 [1]. CD is associated with higher morbidity and mortality compared to vaginal deliveries, including both well-known complications in subsequent pregnancies such as adherent placenta and uterine rupture [2] and less well-known complications such as cesarean section scar pregnancy (CSP).

CSP is defined as a gestational sac that has implanted in the scar of a previous CD [3]. Incidence of CSP is unknown but estimated at 1 in 2000 pregnancies and accounts for 6% of ectopic pregnancies among women with prior CD [4]. Diagnosis is made by sonographic visualization of a gestational sac at the site of the previous CD in the presence of an empty uterine cavity and cervix, as well as thin myometrium adjacent to the bladder [4]. CSP is highly dangerous and an early form of morbidly adherent placenta. The progressive invasion of the placenta is life-threatening and may lead to severe obstetrical complications such as profuse hemorrhage, uterine dehiscence, or rupture [4].

Current management strategies for CSP are based on single case reports or case series, but no standardized diagnostic or management guidelines have been implemented. Moreover, limited literature and data exist on cesarean scar ectopic cases in the second trimester. The aim of this paper is to present a case of a cesarean section ectopic pregnancy diagnosed in the second trimester and perform a literature review for guidelines of management for CSP in the second trimester.

## 2. Case Report

A 35-year-old P1122 presented at 13w4d to our facility from an outside OB/GYN for a second opinion regarding CSP with suspected placenta accreta. The patient reported brown spotting since 7 weeks gestation with lower abdominal pain starting at 12 weeks gestation. The patient reported a history of two prior cesarean sections due to nonreassuring fetal heart tracing for her first pregnancy and uterine rupture during preterm labor for second pregnancy. The patient's medical history included obesity (BMI 32), chronic headaches, hypothyroidism, and polycystic ovary syndrome. Her surgical history included cesarean section, dilation and curettage, and gastric sleeve.

At presentation to our facility, her vitals were within normal limits. Physical exam was notable for a small amount of dark brown blood in the vault and a closed and long cervix with no active bleeding. Laboratory testing including hematocrit (Hct) were within normal limits. Quantitative beta-HCG was 93,345 mIU/mL.

**Figure 1:**
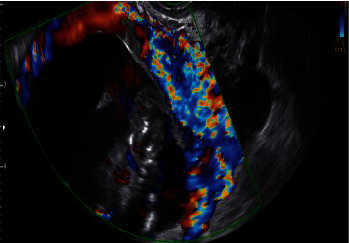
Ultrasound imaging with radiology report noting thickening of the endometrium at the fundus. The gestational sac appears at the lower segment with bulging towards the bladder. In the uterus, there is significant vascularity surrounding the gestational sac and there appears to be very little myometrium.

**Figure 2:**
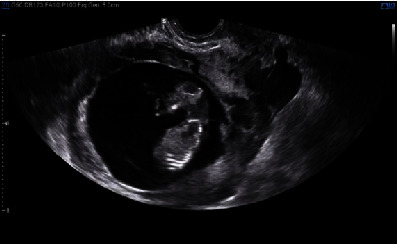
Ultrasound imaging with radiology report noting thickening of the endometrium at the fundus. The gestational sac appears at the lower segment with bulging towards the bladder. On the transvaginal area, there is significant vascularity surrounding the gestational sac and there appears to be very little myometrium.

**Figure 3:**
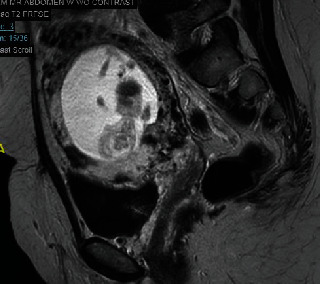
MRI imaging with radiology report eccentric intrauterine pregnancy associated with the c-section scar, extending into the posterior-lateral left adnexa where the placenta is uncovered from the myometrium. The placenta abuts the bladder without evidence for direct invasion.

**Figure 4:**
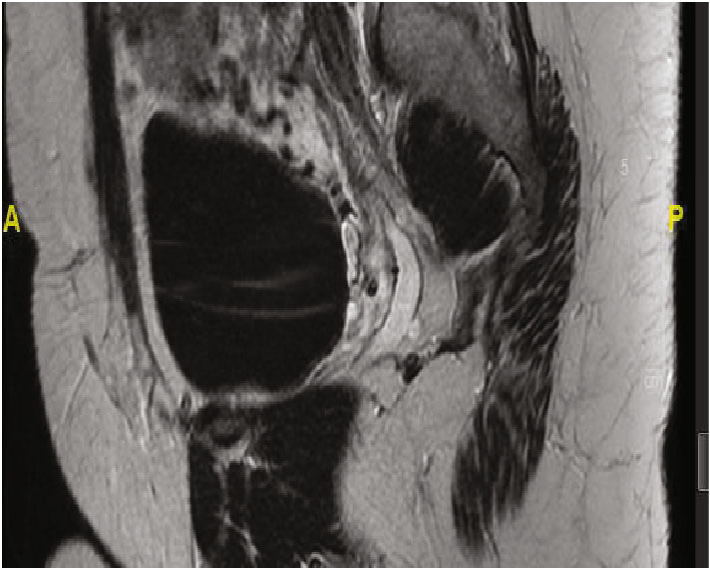
MRI imaging with radiology report eccentric intrauterine pregnancy associated with the c-section scar, extending into the posterior-lateral left adnexa where the placenta is uncovered from the myometrium. The placenta abuts the bladder without evidence for direct invasion.

A transvaginal ultrasound was performed demonstrating a viable singleton pregnancy consistent with 13 weeks and 4 days. The gestational sac was at the lower segment of the uterus, bulging towards the bladder (Figure 1). Significant vascularity was seen surrounding the gestational sac and very little myometrium (Figure 2). An MRI demonstrated a gestational sac with a fetus, which was not contained within the endometrium. Further, the MRI revealed that this eccentric intrauterine pregnancy was associated with the cesarean section scar extending into the left adnexa where the placenta was uncovered from the myometrium (Figure 3). The placenta was abutting the bladder without evidence for direct invasion (Figure 4). The final impression suggested cesarean section ectopic with placenta increta/percreta.

At this juncture, the patient was recommended and accepted inpatient admission for monitoring and preoperative coordination. Gynecology Oncology was consulted for presurgical planning and to perform her surgery given the concern for adherent placenta. Interventional Radiology was also consulted for preprocedural uterine artery embolization to minimize intraoperative blood loss. Urology was available for backup if needed pending the degree of bladder invasion, and Anesthesia was made aware for coordination of availability of blood products. Preoperative Hct was 29.0%. Patient underwent a total laparoscopic hysterectomy with bilateral salpingectomy, bilateral ureterolysis, and cystoscopy. At the time of operation, a 14-week-size uterus was visualized with the pregnancy noted to be emanating from the cesarean section scar. The procedure was uncomplicated, and the patient did well postoperatively. Pathology was consistent with a cesarean scar ectopic pregnancy with a placenta increta. Patient was discharged on the third postoperative day (POD3) and was healing well at her 2-week postoperative check.

## 3. Discussion

CSP is the rarest form of ectopic pregnancy and is uncommonly diagnosed in the second trimester. Given the scarce literature regarding treatment of uterine scar implantation, there is no gold standard of treatment. The management of the CSP is highly variable and borrowed from conventional treatments of abnormal uterine and extrauterine gestation. Management options in the first trimester may differ between providers but pose less challenges due to the small gestational sac and fetus. Past approaches include systemic and local injection with methotrexate, local potassium chloride (KCl) injection, hysterectomy, dilation and curettage, open or laparoscopic scar resection, aspiration, embolization, and hysteroscopy. These options, while documented successfully in early pregnancies, have not been explored in pregnancies diagnosed in later gestational ages.

Our patient posed many challenges due to her late presentation and suspected placentation abnormality at the time of diagnosis. Based on the International Federation of Gynecology and Obstetrics (FIGO) consensus, our patient had a grade 3C on the placenta accreta spectrum defined as abnormally invasive placenta which may reach surrounding pelvic tissues, vessels, and organs [5]. Given these findings and her late gestational age, it was felt that the risks of expectant or medical management were too high. In a meta-analysis looking at 17 different studies of expectant management in patients with CSP resulted in high burden of maternal morbidity including severe hemorrhage, early uterine rupture, and hysterectomy [6]. ACOG, SGO, and SMFM guidelines emphasize the importance of preoperative optimization for accreta spectrum disorders for which a multidisciplinary approach was taken, coordinating between the Gynecology Oncology, Interventional Radiology, Urology, and Anesthesia services. Once discussing surgical options, the decision to attempt a minimally invasive approach was done in order to benefit the patient postoperatively, but consent was done for both minimally invasive and open approaches with both instrument trays present in the room.

Laparoscopic approach in our case presented significant advantages. In addition to obvious benefits of a minimally invasive surgery associated with reduced surgical morbidity and earlier recovery compared to an open procedure, better visualization and magnification afforded by laparoscopic camera allowed for meticulous dissection and an early vascular control of the uterus by opening pelvic sidewalls and dissecting uterine arteries first. Increased intra-abdominal pressure from CO_2_ gas insufflation allowed to significantly minimize the blood loss during the dissection of the protruding gestation sac while preserving the ureters and the bladder.

To better counsel patients and gain a better understanding of the second trimester CSP, a literature review was performed to search for all case reports and case series reporting on second trimester CSP. “Second Trimester Cesarean Section Ectopic Pregnancy” was entered into PubMed, and the results are shown in Table 1. As demonstrated by this search, the information is scarce and the published material on management of second trimester CSP is minimal.

The earliest case report, published in 2006, reported on a patient who underwent expectant management of CSP diagnosed at 16 weeks gestation. The outcome of this case report noted a uterine rupture, during which the uterus was able to be preserved [7]. The second case series, written by Dickerhoff et al., demonstrated three different second trimester CSP, all of which underwent D&C. Two out of three patients had hysterectomies due to uncontrollable hemorrhage and one patient had a hematometra which required reevacuation. This series concluded the high complication rates associated with D&C for CSP and recommended against this treatment modality [8]. The most recent case report described by Sroussi et al. was published in 2017. This case reports discussed a CSP that was diagnosed at 16 weeks. This patient received a preventive pelvic artery embolization after which she underwent a posterior hysterotomy with conservative management of the placenta. No placental remnants were noted at 7 months postop, and the patient had an office hysteroscopy demonstrating a normal uterine cavity [6]. While this case had a successful outcome, consequences of this treatment remain uncertain and application of this treatment to patients with abnormal placentation is unknown.

All in all, management guidelines are scarce for CSP, especially those diagnosed during the second trimester. Our case emphasizes the importance of early diagnosis to facilitate treatment options. Our approach to managing our patient involved a minimally invasive approach with the lowest risk possible for the patient. Additional research and guidelines are needed for this rare pathology which is expected to become more common in the future given the rise of CDs.

## Figures and Tables

**Table 1:** Review of the literature for second trimester ectopic pregnancy.

Reference	GA of presentation	Number of cases in the 2^nd^ trimester	Initial treatment modality used	Outcome
Sroussi et al., 2018 [8]	16	1	MTX, UAE, and posterior isthmic hysterotomy with conservative management of the placenta	Hysteroscopy at 7 months with normal uterine cavity

Dickerhoff et al., 2015 [7]	13	3	D&C	Hematometra
	13			Hysterectomy due to hemorrhage
	14			Hysterectomy due to hemorrhage

Smith et al., 2007 [9]	16	1	Expectant management	Uterine rupture that was preserved during surgery

## Data Availability

There is no underlying data.
